# Dynamic service area sizing in urban delivery

**DOI:** 10.1007/s00291-022-00666-z

**Published:** 2022-02-09

**Authors:** Marlin W. Ulmer, Alan Erera, Martin Savelsbergh

**Affiliations:** 1 Otto-von-Guericke Universität Magdeburg, 39106 Magdeburg, Germany; 2 Georgia Institute of Technology, Atlanta, GA 30332 USA

**Keywords:** Instant delivery, Meal delivery, Service area sizing, Dynamic vehicle routing, Uncertain demand

## Abstract

We consider an urban instant delivery environment, e.g., meal delivery, in which customers place orders over the course of a day and are promised delivery within a short period of time after an order is placed. Deliveries are made using a fleet of vehicles, each completing one or more trips during the day. To avoid missing delivery time promises as much as possible, the provider manages demand by dynamically adjusting the size of the service area, i.e., the area in which orders can be delivered. The provider seeks to maximize the number of orders served while avoiding missed delivery time promises. We present three techniques to support the dynamic adjusting of the size of the service area which can be embedded in planning and execution tools that help the provider achieve its goal. First, we learn the functional dependency between expected demand and the service area that can be supported with the fleet of vehicles. Second, we use value function approximation to improve an initial service area sizing plan for the day based on expected demand. Finally, we introduce a correction mechanism to dynamically adjust the service area sizing plan in response to observed realized demand. Extensive computational experiments demonstrate the efficacy of the techniques and show that dynamic sizing of the service area can increase the number of orders served significantly without increasing the number of missed delivery time promises.

## Introduction

Urban delivery services, especially same-day and instant delivery services, are becoming widespread and commonplace. Retailers like Amazon, Walmart, and Target offer same-day delivery for a growing product selection (Keyes 2019), online delivery platforms like GoPuff provide delivery of convenience store items (Feliciano Reyes 2018), and, most prominently – in part due the COVID-19 pandemic, meal delivery companies and platforms like Domino’s, Grubhub and DoorDash offer instant delivery to satisfy hungry diners (Shead 2019). All of these services have a common feature: customers order during the course of the day and are promised fast delivery; in the case of instant delivery, the promise is very fast (for example, 40 minutes after the time the order is placed in meal delivery). For the actual delivery, service providers typically employ a fleet of drivers (Dai and Liu 2020). Drivers repeatedly perform trips from facilities (fulfillment centers, warehouses, stores, restaurants, etc.) delivering orders to sets of customers. Demand in these environments is uncertain and volatile. Orders only become known when they are placed and order volume varies during the day (e.g., more orders arrive during the lunch and dinner peaks in a meal delivery environment). Furthermore, order volumes can vary from day to day (Littman 2019). As a consequence, ensuring that orders are delivered when promised *and* that drivers are highly utilized is extremely challenging.

Too many missed delivery promises results in dissatisfied customers who may decide no longer to use the service, an issue especially important in meal delivery (Stoll 2019). However, having too many underutilized drivers is costly as they are paid without generating revenue. Three strategies can be considered when seeking to reduce missed delivery promises or to increase driver utilization: better managing demand, better managing drivers, or altering service offerings. In this research, we focus on the first – better managing demand. A common approach to managing demand in the context of urban delivery is to properly define a *service area*, i.e., the area where you are offering to make deliveries (Yildiz and Savelsbergh 2019). Ideally, the service area is chosen large enough to capture many orders but small enough to serve those orders cost effectively (i.e., achieve a high utilization of the fleet of drivers). For the remainder and for ease of presentation, we assume that deliveries are made from a single facility (restaurant) and that the service area is characterized by a maximum travel time (or radius) from the facility, i.e., the maximum time we are willing to travel to reach the location where an order must be delivered.

It is common practice to define a service area radius once and then use that radius every day and at every time during the day. Not surprisingly, in meal delivery environments, this can result in missed delivery promises during the lunch and dinner peak (when the number of placed orders is large) and in underutilized drivers before the lunch peak, in between the lunch and dinner peak, and after the dinner peak (when the number of placed orders is low). Furthermore, companies can experience significant day-to-day variations, for example, the number of meal delivery orders placed increases when the weather unexpectedly worsens (Littman 2019). This suggests that dynamically adjusting the radius in response to observed demand and in anticipation of future demand may be more effective than a single radius. Meal delivery platforms have recently started experimenting with dynamic radii (private communication, instant delivery companies in the USA and Europe, 2021).

Such a dynamic decision environment can be summarized as follows. Throughout the day, customers visit the delivery service’s website to place an order. Based on the customer’s address and the active radius, the service provider decides whether the customer is located in the service area and eligible to place an order. If so, and if the customer does place an order, the customer is promised a latest delivery time. Because all customers within the service area can place an order and all these orders will be delivered, it may not be possible to meet the promised delivery time for all customers. To manage service (missed delivery time promises) and profit (driver utilization), the provider dynamically adjusts the service area radius based on predicted and observed demand. The goal is to find a feasible decision policy that maximizes the expected number of orders, where a policy is feasible if the average delay, i.e., the difference between actual delivery time and promised delivery time, if positive, is less than a threshold.

Some companies we are in discussions with initially considered dynamic service area adjustments as a mechanism to increase market share by guaranteeing service in a certain area (as they do currently) but offering service in a larger area when order volume and delivery capacity allow them to do so. Such an environment may be acceptable, even welcomed, by those customers that did not qualify for home delivery before, but may now receive home delivery at (off-peak) times when the capacity for doing so is available. Meal delivery platforms have recently started exploring this idea (private communication, instant delivery companies in the USA and Europe, 2021).

In both cases, an effective policy should accommodate the expected demand pattern, but also react to deviations from this expected demand pattern. For example, if at a particular time of the day the expected demand is low, the radius should be large enough to ensure high driver utilization. Or, if at a particular time of the day the demand is expected to go up, the radius should be decreased to ensure that future delivery time promises will be met. If deviations to the expected demand pattern occur, the radius should also be adjusted. For example, the radius may be decreased when demand is higher than expected to avoid delays, and the radius may be increased when demand is lower than expected to avoid underutilized drivers.

To accommodate an expected demand pattern and to react to deviations from this expected demand, we propose to combine a number of techniques. First, we use the concept of continuous approximation (CA, Daganzo 1984) to derive a function that takes an expected demand as argument and returns an appropriate service area radius given the fixed delivery fleet. The function is derived by solving many instances with a constant demand pattern and a fixed service area radius. The derived function is then used to determine an initial policy by taking the actual expected demand pattern, partitioning the day into a number of short time periods, and using the derived function to determine a service area radius in each of these time periods based on the expected demand in that time period. To capture the interdependencies of adjacent time periods, we use the concept of value function approximation (*VFA*), a reinforcement learning method (Powell 2011). We use *VFA* to explore policies in the neighborhood of the initial policy. As a result, the radius in one time period may be increased or decreased in anticipation of demand in subsequent time periods. Finally, we develop and embed an day-of-execution correction mechanism to adjust the radius based on the observed demand on a specific day if it deviates from the expected demand. For example, it may increase the radius when the observed demand is low compared to the expected demand. To determine the corrections, the mechanism again relies on *CA*.

We analyze the performance of our proposed approach for determining an effective radius adjustment policy in a comprehensive computational study. The computational study confirms that accommodating expected demand patterns (planning) as well as reacting to deviations from expected demand patterns (execution) can significantly increase performance: Dynamically adjusting the service area radius can increase the number of customers serviced by more than 20% compared to a using a single radius the entire day. Not surprisingly, the higher the demand volatility, the greater the importance of a day-of-execution correction mechanism. However, even without day-of-execution corrections, a few radius adjustments over the course of the day can significantly improve performance.

Our research makes the following contributions:

1. We provide one of the first analyses of the value of controlling the service area size in urban instant delivery services. To this end, we model the problem as a dynamic decision process.
2. We propose a novel approach for producing an effective decision policy that integrates planning and execution controls. While some of the individual components of our approach have been used previously in other planning contexts, integrating day-of-execution corrections is new (and, as our computational study shows, beneficial).
3. We conduct a comprehensive computational study, using restaurant meal delivery as the setting, which clearly demonstrates the benefits of dynamically sizing the service area—the number of orders served increases significantly, by more than 20% in certain cases.

Even though our approach is tailored to and our computational study is restricted to meal delivery, we believe that our ideas can be applied and be valuable in other urban logistic settings, especially when demand is volatile and varies over time, e.g., same-day delivery of goods and dial-a-ride services.

The remainder of the paper is organized as follows. In Sect. 2, we provide a literature review. In Sect. 3, we present the mathematical model. In Sect. 4, we outline our approach for producing an effective decision policy. In Sect. 5, we analyze the results of an extensive computational study. Finally, in Sect. 6, we conclude with final remarks.

## Literature

In the following, we give an overview of the related literature. Our work focuses on the fields of dynamic (meal) delivery routing and service area sizing.

The literature on dynamic delivery routing is summarized in Table 1. We differentiate the literature based on problem characteristics and whether or not demand management is considered.

The problem we focus on is a dynamic routing problem with many vehicles ($\ge 5$) and customers ($\ge 200$), delivery from a warehouse (restaurant), and delivery time commitments. Thus, we classify the problems considered in the literature based on these four characteristics (fleet size, number of customers, delivery from one or more warehouses, and delivery time commitments). If a characteristic is present we indicate that with “$\checkmark$”, and if a characteristic is “partially” present we indicate that with “$(\checkmark )$”. For example, “$(\checkmark )$” in the *Large fleet* column indicates that more than one vehicle is used for delivery but fewer than 5.

The decisions we focus on relate to demand management, i.e., a service area sizing plan and dynamic sizing adjustments during execution. The techniques we use are continuous approximation and value function approximation, a reinforcement learning method. Thus, we classify the solution approaches presented in the literature based on whether demand management is considered and, if so, when, i.e., not at all, as part of planning, as part of execution, or as part of planning and execution. We also list how demand is managed, i.e., service area size or time slots (controlling time slot availability or time slot pricing for customers) and the techniques used to do so, i.e., by enumeration (enum.), by deriving a functional dependency (funct.), by lookahead methods using sampled scenarios (LA), or by reinforcement learning (RL).

**Table 1:** Literature classification

	Problem				Demand management		
Literature	Large Fleet ($\ge 5$)	Many Customers ($\ge 200$)	Delivery	Time Commitment	Planning	Execution	Procedure
Ulmer and Thomas (2018)	($\checkmark$)	$\checkmark$	$\checkmark$	$\checkmark$	($\checkmark$)		Radius (enum.)
Yildiz and Savelsbergh (2019)	($\checkmark$)	$\checkmark$	$\checkmark$	$\checkmark$		$\checkmark$	Radius (funct.)
Gendreau et al. (1999)	($\checkmark$)			$\checkmark$			
Bent and Van Hentenryck (2004)	$\checkmark$		$\checkmark$	$\checkmark$			
Hvattum et al. (2006)	($\checkmark$)					$\checkmark$	Slotting (LA)
Ichoua et al. (2006)	($\checkmark$)	$\checkmark$	$\checkmark$	$\checkmark$			
Thomas (2007)							
Ghiani et al. (2009)	$\checkmark$	$\checkmark$	$\checkmark$	$\checkmark$			
Meisel (2011)						$\checkmark$	Slotting (RL)
Azi et al. (2012)	($\checkmark$)	$\checkmark$	$\checkmark$	$\checkmark$		$\checkmark$	Slotting (LA)
Ghiani et al. (2012)							
Ferrucci et al. (2013)	$\checkmark$		$\checkmark$	$\checkmark$			
Klapp et al. (2018a)			$\checkmark$			$\checkmark$	Slotting (LA)
Klapp et al. (2018b)			$\checkmark$			$\checkmark$	Slotting (LA)
Ulmer et al. (2018a)						$\checkmark$	Slotting (RL)
Ulmer et al. (2018b)						$\checkmark$	Slotting (RL)
Ulmer et al. (2019a)			$\checkmark$			$\checkmark$	Slotting (LA + RL)
Ulmer et al. (2019b)			$\checkmark$			$\checkmark$	Slotting (RL)
Voccia et al. (2019)	($\checkmark$)		$\checkmark$	$\checkmark$		$\checkmark$	Slotting (LA)
Ulmer (2020)	($\checkmark$)		$\checkmark$	$\checkmark$		$\checkmark$	Pricing (RL)
Ulmer et al. (2021)	$\checkmark$	$\checkmark$	$\checkmark$	$\checkmark$			
Chen et al. (2021)	($\checkmark$)	$\checkmark$	$\checkmark$	$\checkmark$		$\checkmark$	Slotting (RL)
Our research	$\checkmark$	$\checkmark$	$\checkmark$	$\checkmark$	$\checkmark$	$\checkmark$	Radius (RL + funct.)

The first two entries in Table 1 represent the research most closely related to ours, i.e., Ulmer and Thomas (2018) and Yildiz and Savelsbergh (2019). In Ulmer and Thomas (2018), customers request delivery during the day and can be served either by a delivery van or by a drone; in case neither is available, a request is rejected. To decide whether to use a delivery van or a drone to serve a customer request, the authors present a threshold policy based on the travel time between the depot and the customer. If the travel time exceeds the threshold radius, then the customer is served by a drone, otherwise the customer is served by a delivery van. An enumeration procedure is used to determine the threshold. Our research is similar in that a travel time threshold is used to determine whether or not service is offered to a customer. However, our threshold radius is both time-dependent and dynamic. We show, among others, the benefits of using a time-dependent and dynamic threshold rather than fixed threshold. Yildiz and Savelsbergh (2019) analyze how the service area size impacts the profit of a delivery platform providing meal delivery services, where the profit depends on the revenue from customers served and compensation payed to delivery drivers. They derive a functional dependency between the revenue and the service area size and other parameters, such as customer arrival rate, revenue per customer, compensation per delivery and miles traveled, and customer satisfaction. Our research is similar in that we also derive a functional dependency between driver utilization and service area size, which is then used to maximize profits. However, we explicitly incorporate the pattern of expected demand over time and the interdependency of service area sizing decisions over time.

The second part of Table 1 lists research that considers related problems or involves demand management. We see that when large-scale problems are considered, as in Ghiani et al. (2009), Ferrucci et al. (2013) or Ulmer et al. (2021), there is typically no mention of demand management. This research mostly focuses on routing technology that ensures fast, on-time service. Ulmer et al. (2021), for example, develops assignment and routing strategies for meal delivery. Thus, their efforts complement our research.

We find that the vast majority of demand management procedures discussed in the literature involve slotting, i.e., deciding when to offer service to individual customers, which involves determining when customers can be served “efficiently.” When service can be offered efficiently is evaluated either by lookahead (LA) or reinforcement learning (RL) methods. LA methods sample scenarios of potential future arrivals and evaluate these scenarios assuming a customer under investigation is served in a particular slot or is not served in that slot. RL-methods compare the value of problem states by means of repeated learning simulations. Then, the value of the states with and without the new customer is compared to decide if service is offered. Good examples this type of research are Klapp et al. (2018a, b) and Ulmer et al. (2019b), where a single-vehicle setting is investigated and LA and RL methods are employed to decide whether to serve a customer request or not. In Ulmer (2020), reinforcement learning is used, instead, to decide delivery prices; based on the delivery fee customers decide to complete an order or to walk away. All previous work on demand management focuses on day-of-execution and individual customers, usually for settings with one or possibly a few vehicles and a small number of customers, whereas our work extends to planning and sets of customers.

Besides dynamic delivery routing, service area considerations also appear as part of demand management strategies in attended home delivery. In these environments, order acceptance and order delivery are clearly separated. That is orders are accepted over a period of time, but the routing of delivery vehicles only occurs after the order acceptance phase has finished. Some research in this area proposes to use so-called resource buckets for areas of the service region (see for example (Cleophas and Ehmke 2014)). Once the number of accepted orders in a resource bucket exceeds its capacity, service in that area is no longer offered. Our setting is different, because new customers arrive while orders that were placed earlier are already being delivered. Thus, resource management is more time-critical and immediate.

For further reading on other related topics, we suggest Strauss et al. (2018) and Klein et al. (2020) for revenue and demand management, Pillac et al. (2013) and Ulmer et al. (2020) for dynamic vehicle routing, and Alnaggar et al. (2021), Sampaio et al. (2019), and Savelsbergh and Ulmer (2022) for crowd-sourced delivery.

## Problem definition

In the following, we define the problem and present the mathematical model. We first give a problem narrative. We then describe the dynamic decision process.

### Problem narrative

We consider a service provider delivering items from a facility to customers with a delivery time promise. Even though this captures a variety of settings, for ease of presentation, we focus on a meal delivery environment.

In a meal delivery setting, customers open the provider’s web page and enter their address to find out if they are eligible for a delivery. A customer’s eligibility to receive a delivery depends on their location. Eligible customers that place an order are promised that delivery will take place within a certain amount of time, e.g., within 40 minutes after the order is placed. The delivery time promise is the same for every customer. Order placement is a stochastic process and order placement volume can vary by day and by time of day. In meal delivery, the number of orders during the week tends to be higher than the number of orders during the weekend and order placement peaks during lunch and dinner hours (Dai and Liu 2020). Furthermore, in meal delivery, order volume is impacted by special events and weather conditions (Littman 2019). This indicates that the stochastic process representing order placement is not memoryless. For the delivery of orders, the provider uses a fixed fleet of vehicles (operating during the period that orders can be placed and some time after that to complete final deliveries). The provider seeks to maximize the expected number of orders served (as a proxy for profit) while ensuring a target service level. For example, in meal delivery, late deliveries do not only result in customers waiting but also less fresh food. Thus, the provider may seek to have small delivery lateness (or delay) over all served customers, for example, less than 1 minute on average per customer (this will be modeled as a chance constraint).

To achieve the desired service level, the provider manages the size of the service area. More specifically, at a fixed set of decision epochs during the day, the provider sets a service area radius around the facility (restaurant) to be used until the next decision epoch. Customers within the service area, i.e., with a travel time between restaurant and customer that is less than the radius, are eligible to receive a delivery; customers outside the service area, i.e., with a travel time between depot and customer that is larger than the radius, are ineligible and cannot place an order. The sizing decision is informed by the observed customers so far and the expected customers for the remainder of the day. Placed orders are delivered by the vehicles in the fleet on a trip that starts and ends at the facility; a vehicle typically makes multiple trips per day. We assume that the assignment of orders to vehicles and the routing of the vehicles are performed by a predefined assignment and routing policy, i.e., these decisions are not under our control. More specifically, we assume that the provider only observes demand (eligible and ineligible) and observes realized delivery lateness (delay).

### Stochastic dynamic decision process

In the following, we present a mathematical model of the meal delivery setting. Because order placements are stochastic and decisions are made dynamically, we model the setting as a stochastic dynamic decision process following Powell (2011). A stochastic dynamic decision process consists of the following components: decision points, decision states, decisions, reward function, state transition function, and exogenous information. We start by defining the parameters and notation and by giving an illustration of the system dynamics. We then define the various model components.

#### Preliminaries

We assume that a fleet of *m* vehicles delivers orders placed at time points during the planning horizon ${\mathcal {T}}=[0,1,\dots ,T]$. For the remainder, we will assume regularly spaced time points one time unit apart (e.g., a time point at every minute of the day). The vehicles start and end their shift at facility *D* (a shift ends when a vehicle has served all orders assigned during the planning horizon). The travel time between two locations, $l_1$ and $l_2$, is given by function $\tau (l_1, l_2)$. The time to load items into a vehicle at the facility is given by $\tau ^D$ and independent of the number of items to load. The time to deliver an item at a customer is $\tau ^C$ and independent of the customer. We assume travel, loading, and delivery times are a multiple of the time unit (thus are integer valued). The facility and customers are located in an area ${\mathcal {A}}$. The location of *D* is denoted by $a_D \in {\mathcal {A}}$ and that of a customer *C* by $a_C \in {\mathcal {A}}$. The time a customer *C* arrives, i.e., attempts to place an order, is denoted by $t_C \in {\mathcal {T}}$ (recall that depending on the active service area radius, a customer may be ineligible to place an order). The customer arrivals are a stochastic process with memory. The delivery plan, i.e., the assignment of orders to vehicles and the routing of the vehicles, is updated whenever a new order is placed. In our experiments, we use the fast assignment and routing heuristic presented in Ulmer (2017). This greedy heuristic examines all (partial) vehicle routes to identify the insertion position that minimizes additional delay, and, as a tiebreaker, minimizes additional travel time. We note that our approach for determining an effective policy for deciding the service area radius is independent of the chosen heuristic. However, we do require that the time that an order of customer *C* is delivered, denoted by $t^d_C$, is revealed as soon as it occurs. The delivery time promise is $\delta$, i.e., if customer *C* places an order at $t_C,$ the delivery time promise is $t_C + \delta$, and the customer experiences a delay if $t^d_C > t_C + \delta$. We denote the delay for customer *C* by $d_C$ and set $d_C=\max \{0,t^d_C-(t_C+\delta )\}$.

#### Illustration of system dynamics

**Fig. 1:**
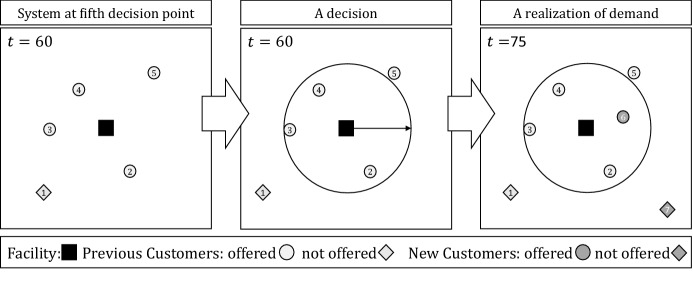
Example for a state, a decision, and a realization of stochastic information

Before we introduce the components of the dynamic decision process in detail, we illustrate the system dynamics with a small example. For ease of presentation, we omit information on the vehicles delivering orders and any delay experienced by customers. We assume that sizing decisions are made every 15 minutes. Figure 1 shows the system at the fifth decision point (left part), a decision (middle part), and a realization of demand during the next 15 minutes (right part). The facility (restaurant) is represented with a black square, customers that were offered delivery are represented by circles, and customers that were not offered delivery by diamonds.

At the fifth decision point, one hour has already passed and four radius decisions have already been made. Five customers have attempted to place an order; Customer 1 was deemed ineligible at the time of the attempt and Customers 2 thru 5 were deemed eligible at the time of their attempt. Based on this information and information about expected future orders, the provider decides the service area radius to use for the next 15 minutes. This decision controls whether or not a customer attempting to place an order in the next 15 minutes will be successful or not. Two new customers attempt to place an order in the next 15 minutes. The delivery location of Customer 6 is within the active service area and the customer can place an order; the delivery location of Customer 7 is outside the active service area and cannot place an order. Thus, the realized reward between $t=60$ and $t=75$ is one, because one additional customer could be offered service. At time 75, the sixth decision point, the provider again decides the radius for the next 15 minutes.

#### Customer arrivals

Decisions regarding the active service area radius are made every ${\bar{t}}$ time units with the first decision at the start of the day. Let the set of decision time points be ${\mathcal {T}}^D = [0,{\bar{t}},2{\bar{t}},\ldots ,(K-1){\bar{t}}]$ with $K{\bar{t}} = T$ (we assume ${\bar{t}}$ divides *T*). Thus, a day is partitioned into time intervals $[(k-1){\bar{t}}, k{\bar{t}})$ for $k=1,\ldots ,K$. For each interval $[k{\bar{t}}, (k+1){\bar{t}})$, let ${\mathcal {C}}_{k+1}$ be a random variable denoting the set of customers arriving in the interval and let ${\mathcal {C}}^\omega _{k+1}$ for $\omega \in \Omega _{k+1}$ denote a specific realization of customer arrivals in the interval where $\Omega _{k+1}$ is the set of all possible customer arrivals in the interval. Because we model customer arrivals as a stochastic process with memory, we have that $\Omega _{k+1}$ depends on $\Omega _{k}$. The combination of all possible realizations of customer arrivals in the intervals leads to a set of possible realizations for the entire day, i.e., $\Omega = \prod _{k=0}^{K-1} \Omega _{k+1}$. Finally, let ${\mathcal {C}}_{0k}$ denote an observed set of customer arrivals in the interval $[0,k{\bar{t}})$ for $k=1,\ldots ,K$.

#### State variables

A state $S_k$ is defined by a decision time point, $k {\bar{t}}$, and observed customer arrivals up to that time point, ${\mathcal {C}}_{0k}$, i.e., a list of $n_k$ customers $C_1, \ldots , C_{n_k}$ with for each customer its location and the time at which the customer arrived. Thus, a state can be represented as $S_k = (k, {\mathcal {C}}_{0k})$ with the initial state being $S_0 = (0, \emptyset )$.

#### Decision variables

A decision $x_k \in X(S_k)$ at decision time $k{\bar{t}}$ sets the active service area radius for the time interval $[k{\bar{t}}, (k+1){\bar{t}})$. The (immediate) reward $R(S_k,x_k)$ of a decision $x_k$ in state $S_k$ is a random variable and represents the expected number of customers arriving in the time interval $[k {\bar{t}},(k+1) {\bar{t}})$ with a location within the active service area, i.e., the expected number of order placements. Therefore, the reward function is

1 $$\begin{aligned} R(S_k,x_k)={\mathbb {E}}\left[ \left( \left| \{C \in {\mathcal {C}}^\omega _{k+1}: \tau (a_D,a_C)\le x_k\}\right| \right) |S_k\right] . \end{aligned}$$

Recall that the we assume that customer arrivals are a stochastic process with memory, thus the potential realizations ${\mathcal {C}}^\omega _{k+1}$ and the expected reward depend on the observed demand which is captured in state $S_k$.

#### State transitions

Once a decision has been made, a realization of customer arrivals ${\mathcal {C}}^\omega _{k+1}$ for the time interval $[k {\bar{t}}, (k+1) {\bar{t}})$ is observed. The leads to a state $S_{k+1}$ at time $(k+1) {\bar{t}}$ with observed demand ${\mathcal {C}}_{0,k+1} = {\mathcal {C}}_{0k} \cup {\mathcal {C}}^\omega _{k+1}$.

#### Objective and service level

A solution to the problem is a policy $\pi$ from among the set of policies $\Pi$. A policy $\pi$ maps each state $S_k$ to a radius decision ${\mathcal {X}}^\pi (S_k)$. An optimal policy maximizes the expected reward

2 $$\begin{aligned} \pi ^*=\mathop {\mathrm{arg\,max}}\limits \limits _{\pi \in \Pi } {\mathbb {E}} \left[ \sum \limits _{k=0}^K{R(S_k, {\mathcal {X}}^\pi (S_k))|S_0}\right] . \end{aligned}$$

One policy is to set the radius to infinity regardless of the state. In that case, all arriving customers can place an order and are served. However, many customers will experience a substantial delay. Thus, we restrict the set of policies by means of a chance constraint, namely that the expected average delay per customer must lie below a threshold $L \ge 0$.

To this end, we define a quality measure $Q(\pi , {\mathcal {C}}^\omega )$ for a policy $\pi$ and a given realization of customer arrivals ${\mathcal {C}}^\omega$ with $\omega \in \Omega$. The quality measure relates to the observed delay during an entire day. Let ${\mathcal {C}}^{\omega ,\pi }$ be the set of order placements when policy $\pi$ is applied (i.e., the set of customers with a location within the active service area at the time of their arrival). Then, the observed delay is

3 $$\begin{aligned} Q(\pi ,{\mathcal {C}}^{\omega })=\sum \limits _{C \in {\mathcal {C}}^{\omega ,\pi }} d_C. \end{aligned}$$

A policy is considered feasible if the expected delay divided by the expected number of order placements (i.e., the average customer delay) is less than threshold *L*:

4 $$\begin{aligned} \frac{{\mathbb {E}} Q(\pi ,{\mathcal {C}}^{\omega })}{{\mathbb {E}} |{\mathcal {C}}^{\omega ,\pi }|} \le L. \end{aligned}$$

Note that, the average customer delay for a specific realization of customer arrivals can be larger than *L*.^1^

## Solution approach

Finding an optimal policy is challenging because of the dimensions of the state and decision spaces. Thus, we present a heuristic. In the following, we present our solution approach for finding a high-quality policy. We first give a motivation and an overview of the steps taken. We then describe each of the steps in detail.

### Motivation and overview

The design of our policy is driven by three practical considerations:

1. *At times with high expected demand, the service area should be small and at times with low expected demand the service area should be large*. When the number of orders in a given service area increases, the workload per vehicle increases measured by number of deliveries as well as travel distance. Consequently, the likelihood of delays increases. To achieve a desired level of service, the area may need to be reduced. Similarly, when the number of orders in a given service area decreases, the workload per vehicle decreases. Consequently, the likelihood of vehicle idle time increases. To achieve a desired level of vehicle utilization, the area may have to be enlarged.
2. *Deciding the size of the service area should not only consider the expected demand at the time of the decision but also the expected future demand*. The size of the service area affects the percentage of arriving customers that are allowed to place an order. However, orders that are placed are not delivered instantaneously, because it may take time before a vehicle is available at the depot to load the order, to travel to customer location, and to deliver the order. Thus, the size of the service area also impacts the workload of the vehicles in subsequent time intervals and, thus, the ability to deliver orders placed in the future. Therefore expected future demand should be considered when deciding its size.
3. *If the realized demand is higher (lower) than expected, the service area should be smaller (larger) than usual*. When we observe a larger than expected number of customer arrivals, the workload per vehicle will be higher than expected. Thus, to ensure the desired level of service, the service area should be reduced. This is especially true since it is likely that a larger number of customer arrivals will also be observed in the future. Similarly, when we observe a smaller than expected number of customer arrivals, the workload per vehicle will be lower than expected and the service area should be enlarged.

Using these considerations, we develop a heuristic approach with the following features:

1. *Continuous Approximation (CA)*. To incorporate the first consideration, we adapt the idea of Daganzo (1984) and approximate a function that maps a number of customers to the fleet size required to serve these customers. In our case, we derive a function that maps an expected stream of customer arrivals to an “optimal” radius, i.e., a radius that allows the largest number of orders to be delivered without causing an average delay that exceeds the threshold. We derive this function by analyzing combinations of a constant expected customer arrival rate and a service area of a given size and finding the maximum areas that do not exceed the threshold. We then use regression to determine the functional dependency between expected customer arrival rate and maximum service area radius. We use this function to derive time-dependent radii for instances with heterogeneous expected customer arrival rates over time.
2. *Value Function Approximation (VFA)*. To incorporate the second consideration, we search the space of policies around the policy obtained by *CA* with value function approximation (*VFA*, Powell 2011). *VFA* is a reinforcement learning technique seeking to learn the value of decision in a state via repeated simulation and updates. For our problem, the *VFA* approximates the expected reward to go of setting a service area radius at a specific time in the horizon, i.e., the expected number of orders served until the end of the day. Higher than desired average customer delay values are incorporated by means of a penalty term. *VFA* starts with initial values and then repeatedly selects a policy (a set of radii) based on the values, simulates the policy, and updates the approximated values using the observed values. In contrast to the initial policy obtained using *CA*, *VFA* produces a policy that reflects the interdependency of the chosen radii.
3. *Day-of-execution Correction Mechanism*. To incorporate the third consideration, we adjust the radii of the policy produced by *VFA* using recently observed customer arrival rates (to adjust for higher or lower than expected customer arrivals). To do so, we use the function that maps an expected customer arrival rate to a service area radius. Specifically, we take a convex combination of the radius suggested by the policy produced by *VFA* and the radius suggested by the function for the recently observed customer arrival rate.

Combining the concepts of *CA* and *VFA* was already proposed in Ulmer and Savelsbergh (2020) for a tactical workforce scheduling problem, but the implementations of *CA* and *VFA* for this problem setting are new. The integration of day-of-operation corrections is novel and, as we show in our computational study, beneficial. We refer to the policy obtained by using all three techniques as the *anticipatory service area radius sizing policy with correction mechanism* (*ARS*$^+$)^2^. In the following, we describe the algorithmic details of the three techniques.

### Continuous approximation

In the following, we describe how we use *CA* to determine a function that maps an expected stream of customer arrivals to a service area radius.

We generate *M* sets of *H* customer arrival sequences using (constant) expected arrival rates $\nu _m$ for $m = 1,\ldots ,M$. We omit any customers arriving in the last hour of the day to avoid ending effects and denote the resulting sets of customer arrival sequences by $\Omega ^{m}$, $m=1,\dots ,M$.

Given that customers arrive at a given rate throughout the day, we assume that the same service area radius is used throughout the day as well, i.e., we assume a policy $\pi _i$ mapping each state to the same service area radius *i*. For each set of customer arrival sequences, i.e., $\Omega ^{m}$ for $m=1,\dots ,M$, we determine the maximum service area radius that does not violated the average customer waiting time limit (see Equation (4)). The average customer waiting time is the ratio of accumulated delay over all *H* sequences, with $Q(\pi _i,{\mathcal {C}}^{\omega ^h}$) measuring the observed delay in sequence *h*, divided by the number of served customers in all *H* sequences, with $|{\mathcal {C}}^{\omega ^h,\pi _i}|$ representing the number of served customers in sequence *h* when applying policy $\pi _i$:

5 $$\begin{aligned} \frac{\sum \nolimits _{h=1}^H Q(\pi _i,{\mathcal {C}}^{\omega ^h})}{\sum \nolimits _{h=1}^H |{\mathcal {C}}^{\omega ^h,\pi _i}|}. \end{aligned}$$

We now find the maximum radius by a simple search – increasing the service area as long as the average waiting time violation limit *L* is not exceeded, i.e.,

6 $$\begin{aligned} x^m=\mathop {\mathrm{arg\,max}}\limits \limits _{i \in {\mathbb {N}}} \frac{\sum \nolimits _{h=1}^H Q(\pi _i,{\mathcal {C}}^{\omega ^h})}{\sum \nolimits _{h=1}^H |{\mathcal {C}}^{\omega ^h,\pi _i}|} \le L. \end{aligned}$$

The value $x^m$ is the service area radius selected for expected customer arrival rate $\nu _m$.

In this way, we get policies for each of the *M* expected customer arrival rates, $(\nu _{1},x_{1}), \dots ,$ $(\nu _{M},x_{M})$. We then use regression to fit a power function through these data points. A power function can flexibly model that the radius should decrease as the demand increases (as stipulated by Consideration 2). We obtain a function $\rho (\nu )$ that maps an expected customer arrival rate to a service area radius, i.e.,

7 $$\begin{aligned} \rho (\nu )=a \nu ^{b}, \end{aligned}$$

where the parameters *a* and *b* are determined by the regression.

**Fig. 2:**
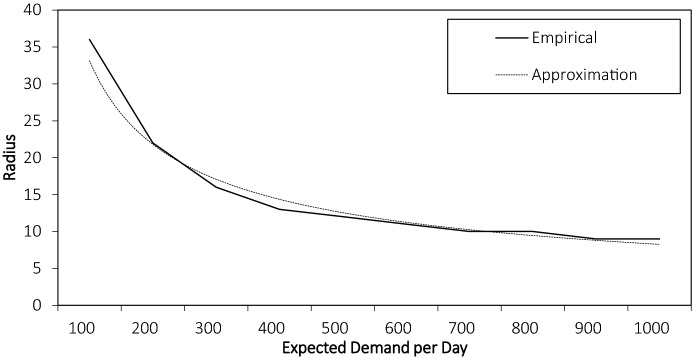
Comparison of empirical radius and power-function approximation for *CA*

In our experiments, we set $M=11$ and $H=500$. We increase the expected number of customer requests per day in steps of hundreds from $\Omega ^{1}$ with expected demand of 100 to $\Omega ^{11}$ with expected demand of 1000 requests per day. For the geographical settings described later in the paper, the corresponding empirical radii and the power-function approximation are depicted in Fig. 2. We observe a monotone decrease in the radius when the demand increases. We further see that a power-function approximation can provide an accurate representation of the observed radius values.

In the setting of interest, expected customer arrival rates are not constant but vary during the day. We use the function $\rho (\nu )$ to determine a policy for this setting as follows. Preliminary tests with setting a service area radius using $\rho (\nu )$ at each decision time point yielded poor results, most likely because it cannot capture interdependencies between radii decisions. Thus, we aggregate time intervals into a smaller set of time periods ${\mathcal {P}} =\{1, \dots , p_\text {max}\}$ each with length $\frac{T}{p_\text {max}}$. In our computational experiments, we have used $p_\text {max} = 4$ with each period *p* representing two hours. Within a time period, the same radius will be set at every decision time point. That is, given expected customer arrival rate $\nu _p$ for time period *p*, we set the service area radius $x_p$ to $\rho (\nu _p)$.

The expected customer arrival rate $\nu _p$ for a period *p* can be determined based on the distribution of request times $t_C$ throughout the day (for example, based on historic observations or predictions). Given a vector of expected customer arrival rates, $(\nu _{1}, \ldots , \nu _{P})$, the service area radii are set to $\left( \rho (\nu _{1}), \ldots , \rho (\nu _{P})\right)$. Because this policy may not be feasible or unnecessarily conservative, we do some fine tuning. We adjust the radii in a period as follows:

8 $$\begin{aligned} {\widehat{x}}_p = \left\lfloor \epsilon \times \rho (\nu _p) \right\rfloor , \end{aligned}$$

where $\epsilon$ is the maximum value such that the corresponding policy does not exceed the average customer delay threshold. Again, we use simulation to identify $\epsilon$. We start with $\epsilon =0$ and increase $\epsilon$ in small steps of 0.05 based on preliminary computational tests. We denote the resulting policy by *CA*.

### Value function approximation

The obtained *CA*-policy provides radii based on the expected demand in the corresponding period. However, it fails to consider the interdependencies of radius-decisions in different periods.

As Ulmer and Savelsbergh (2020), we use *VFA* to search the space of policies around *CA* to capture temporal interdependencies, e.g., an expected increase or decrease in customer arrivals. In the following, we define the value function for the problem at hand, we describe the solution space being searched, and we present the learning procedure.

For a state $S_k$ and each possible decision $x_k \in X_k(S_k)$, the value function *V* defines the expected reward to go when taking the decision that assuming an optimal policy $\pi ^*$ used for the remainder of the day:

9 $$\begin{aligned} V(S_k,x_k)= {\mathbb {E}} \left[ R(S_k, x_k) + \sum \limits _{l=k+1}^K R(S_l, {\mathcal {X}}^{\pi ^*}(S_l))|S_k, x_k\right] . \end{aligned}$$

The value function provides a value for each pair of state and decision. In our *VFA*, we approximate the values by means of simulation. Because of the vast number of state-decision pairs, we aggregate the states. Instead of the full state information, we solely focus on the time and ignore the observed customer arrivals. As before, we also aggregate the time intervals into time periods $p = 1, \dots , P_\text {max}$. We consider the full decision space. Thus, our approximate value function ${\widehat{V}}$ provides the value ${\widehat{V}}(p,x_p)$ of a service area radius decision $x_p$ at the beginning of period *p* and we use simulation to approximate the values of ${\widehat{V}}$.

To this end, we define an area of the space of policies that *VFA* searches. Specifically, we create a neighborhood around policy *CA* as follows. For each period *p*, we consider radii in the range

10 $$\begin{aligned} X^\gamma _p= \left\{ \left[ \left\lfloor (1-\gamma ) x^{ {CA}}_p\right\rfloor \right] ^+, \left[ \left\lfloor (1-\gamma ) x^{ {CA}}_p\right\rfloor +1 \right] ^+, \ldots , \left\lceil (1+\gamma ) x^{{CA}}_p\right\rceil \right\} , \end{aligned}$$

for a $\gamma > 0$, where we use notation $[\cdot ]^+$ to indicate the positive part, i.e., $[x]^+ = \max \{x,0 \}$. Larger values of $\gamma$ and $x^{ {CA}}_p$ result in a larger radius range in a period. This allows for the smoothing of policy *CA*. We also consider a minimum range

11 $$\begin{aligned} X^r_p=\left\{ \left[ x^{ {CA}}_p-r \right] ^+, \left[ x^{ {CA}}_p-r+1 \right] ^+, \ldots , x^{ {CA}}_p+r \right\} , \end{aligned}$$

for an $r \in {\mathbb {Z}}_{>0}$. The union of the two sets $X_p = X^\gamma _p \cup X^r_p$ is the set of considered radii for period *p*. Thus, the search space is $X= X_1 \times X_2 \times \dots \times X_{P}$. Because policy *CA* is in the search space, the search space has at least one feasible policy.

*VFA* systematically searches the defined space by iteratively selecting a policy, simulating the policy, and using the simulation results to update the values of ${\widehat{V}}$. In the following, we give a description of the process (for algorithmic details, see Ulmer and Savelsbergh (2020)).

The first policy selected is $(x_1^{CA},\dots ,x^{CA}_{p_\text {max}})$. Policies in subsequent iterations $i=1, \dots , I$ are selected by means of Boltzmann exploration, as suggested in Brinkmann et al. (2019). Using Boltzmann exploration, the probability of selecting a particular radius decision $x^i_p$ in a period *p* depends on the current value ${\widehat{V}}^i(p,x^i_p)$, the range of current values associated with the possible radii in the period, and the iteration. We denote the policy selected in iteration *i* by $x^i=(x_1^i, \dots , x^i_{p_\text {max}})$.

A selected policy is evaluated by simulating a batch of 1,000 realizations of customer arrivals. The simulation returns the average realized value of the pairs $(p,x_p^i)$, denoted by $v^i(p,x_p^i)$. It also returns the delay *Q* and the delay $Q_p(p,x_p^i)$ for the order placements in periods $p^\prime$ with $p^\prime \ge p$. The latter allows us to penalize parts of the policy, even if the policy itself is feasible.

Both realized values and delays are used to update the values of ${\widehat{V}}$. When the delay $Q_p$ is greater than threshold *L*, we calculate a penalty $\lambda (i+1) (Q_p - L)$ that depends on the *VFA* iteration *i* and a parameter $\lambda$. Tying the penalty to the number of iterations leads to an increasing penalization of infeasible solutions over the iterations, a procedure often observed in heuristic search procedures. The corrected value for state $(p, x^i_p)$ is

12 $$\begin{aligned} v^i(p,x^i_p) = v^i(p,x_p^i) - \underbrace{ \lambda (i+1) (Q_p - L)_{+}}_{\text {Penalty Term}}. \end{aligned}$$

Given this calculated value, the state value is updated as follows:

13 $$\begin{aligned} {\widehat{V}}^{i+1}(p,x^i_p) = (1-\eta ) \times {\widehat{V}}^i(p,x^i_p) + \eta \times v^i(p,x^i_p), \end{aligned}$$

Parameter $\eta$ determines the stepsize of the update process. We set $\eta =\frac{1}{\sqrt{n(p,x^i_p)}}$ with $n(p,x^i_p)$ being the number of observations of $x^i_p$ in period *p*. This results in a stronger emphasis on the value of $v^i(p,x^i_p)$ in later observations when the values becomes likely more accurate (Powell 2011).

We run *VFA* with $I=1000, \; r=2$ and $\lambda =100$. We further explore $\gamma = \frac{1}{2}, \frac{1}{3}$ and $\frac{1}{4}$ and select the policy that performs best. We select a high number of *I* to ensure statistical significance. The values for *r*, $\lambda$, and $\gamma$ are based on preliminary computational tests. We denote the best policy encountered during the search by *anticipatory radius sizing policy* (*ARS*).

### Day-of-execution correction mechanism

Policy *ARS* captures expected customer arrivals as well as temporal and spatial consolidation and should perform well when daily customer arrivals are as expected. However, the policy does not react to observed customer arrivals, i.e., does not adjust to days with fewer than expected customer arrivals or days with more than expected customer arrivals. Such reactions may avoid delays when there are more than expected customer arrivals and may avoid low vehicle utilization when there are fewer than expected customer arrivals.

To allow such reactions, we combine *ARS* with a day-of-execution correction mechanism (CM). The CM observes customer arrivals and adjusts the service area radius accordingly. CM is applied at every decision time point regardless of the state. More specifically, for a state $S_k$, we calculate the customer arrival rate $\nu _k$ in the last $\Delta$ time units

14 $$\begin{aligned} \nu _k = | \{C \in \widehat{{\mathcal {C}}}_k: t_C \ge k{\bar{t}} - \Delta \}| / \Delta . \end{aligned}$$

The decision in state $S_k$ is then

15 $$\begin{aligned} x_k = (1-\alpha ) x_p^{ {ARS}} + \alpha \rho (\nu _k), \end{aligned}$$

with $\alpha \in \left[ 0,1 \right]$ a parameter controlling the emphasis on the correction mechanism.

When a day-of-execution correction mechanism is in use on a daily basis, it should also be in effect when we determine policy *ARS*. Therefore, we integrate CM in *ARS*. *ARS* still learns a vector of radii via *VFA*, however, CM is already applied in the decision-making and evaluation of the value function. Based on preliminary testing, we set the CM-horizon to $$\Delta =30$$ minutes. We test $$\alpha \in \{0.1, 0.2, 0.3, 0.4, 0.5\}$$ and select the best $$\alpha$$ for each instance setting. The best results are usually obtained with $$\alpha$$ between 0.1 and 0.3. We denote the best policy obtained during the policy search when the day-of-execution correction mechanism is active as *ARS plus CM*, $$ {ARS}^+$$.

## Computational study

In this section, we present our computational study. We first describe the instances and the benchmark policies. We then analyze the performance of the various policies.

### Instances and benchmark policies

In the following, we describe the instances and the policies tested.

#### Geography

We assume a central facility in the service area at location (0, 0) and a fleet of 10 vehicles. Each vehicle travels at a speed of 25 kilometers per hour (von Schneidemesser 2015). Service and loading times are set to 2 minutes. The coordinates of the customer locations are iid and follow a normal distribution with mean zero and standard deviation of 2.5 km. This resembles what has been observed in practice, i.e., that the number of customers declines with the distance to the restaurant, possibly because customers worry about the freshness of the delivered food due to the longer travel times. The travel distances are set as Euclidean distances multiplied with factor 1.4 to simulate a street network (Boscoe et al. 2012). In combination with the customer location distribution, this implies that about 99% of the customers are located less than 10 km and 25 minutes travel from the facility. All travel times are rounded up to minutes.

**Fig. 3:**
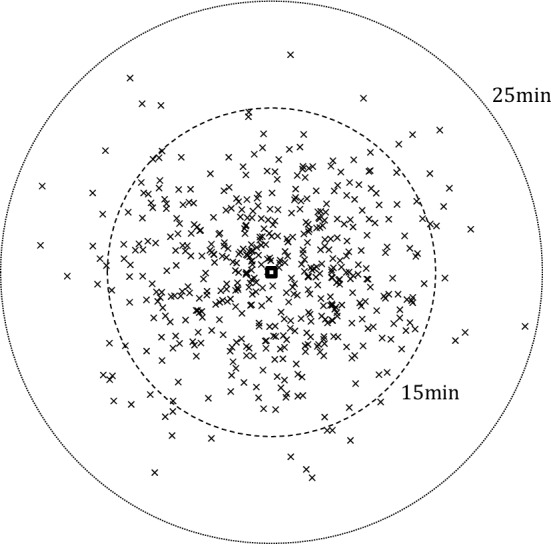
Example for customer locations for one day

An example day of customer locations is depicted in Fig. 3. The facility is shown as the square in the center and each customer location is shown as a cross. The dashed circles are at the radii of 15 and 25 minutes of travel from the facility, respectively. We see that most customer locations are within 15 minutes travel from the facility with a few customers being farther away.

#### Customer arrivals

**Fig. 4:**
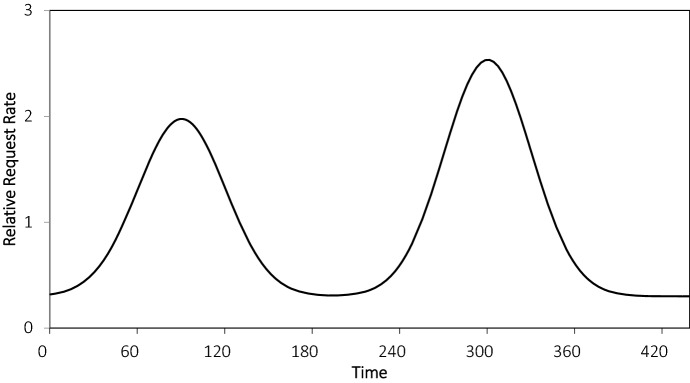
Daily customer arrivals pattern

We assume a horizon of 420 minutes plus sufficient time afterward for the vehicles to deliver the final orders. The service promise to customers is 40 minutes after order placement. Radius decisions are made every 15 minutes. We assume customer arrivals only occur in the first seven hours (so that at least one hour is available for delivery). The expected number of requests per day is 500. We model customer arrivals as a combination of three arrival streams: one base stream, one smaller noon stream, and one larger evening stream. This arrival pattern is common in meal delivery settings, see for example Dai and Liu (2020). All streams generate customer arrivals via Poisson processes. The base stream is homogeneous over time; the other two are nonhomogeneous over time. The noon and evening streams produce customer arrival times that are normally distributed with standard deviation of 30 minutes and means of minutes 90 and 300, respectively. In expectation, 30% (or 150) of the customer arrivals are from the base stream, 30% (or 150) are from the noon stream, and 40% (or 200) are from the evening stream. The combination is shown in Fig. 4. The x-axis depicts time; the y-axis depicts the relative arrival rate. We observe the two peaks around minutes 90 and 300.

To model daily variations, we vary the expected number of customer arrivals per stream. More specifically, we sample the expected number of customer arrivals for a stream from a normal distribution, with mean 150 for the base stream, mean 150 for the noon stream, and mean 200 for the evening stream. Even though the *expected* expected number of customer arrivals for a day is always 500, this procedure leads to days with smaller and larger demand peaks and, therefore, days with fewer or more customer arrivals. These variations can be recognized, and acted upon, by monitoring customer arrivals; the number of customer arrivals early in the day is a predictor of the number of arrivals later in the day. Thus, the customer arrival process is not memoryless. When generating instances, i.e., realizations of daily customer arrivals, we use different coefficients of variation (COV) for these normal distributions: 0.0, 0.1, 0.2, 0.4, and 0.6. With increasing COV, the variance in the number of (daily) customer arrivals increases as well.

We generate 2000 realizations of customer arrivals for each COV value, using 1000 realizations for learning and 1000 realizations for evaluation. Instances are available upon request.

#### Policies

We compare the following policies:

- *FIXED*: The service area is fixed for the entire day with the largest radius possible. This radius is determined by starting from zero and increasing it in small increments until the average customer delay exceeds the threshold.
- *CA*: This policy uses the CA-radii.
- *VFA*: This policy uses the radii learnt when *VFA* is initialized with *FIXED*.
- *ARS*: This policy uses the radii learnt when *VFA* is initialized with *CA*.
- ${ARS}^+$: This policy uses the radii learnt when *VFA* is initialized with *CA* and uses CM during training.

We determine five variants of these policies, one for each of the different COV values.

### Performance

We evaluate each of these policies (i.e., each of its five variants) using 1000 realizations of customer arrivals. For each of the policies, the average customer delay was below the threshold of one minute. Because the service area radius for *FIXED* tends to be small, the average delay is usually smallest for *FIXED*.

All statistics reported in the remainder are averages over the realizations of customer arrivals used in the evaluation (even though not explicitly stated).

**Fig. 5:**
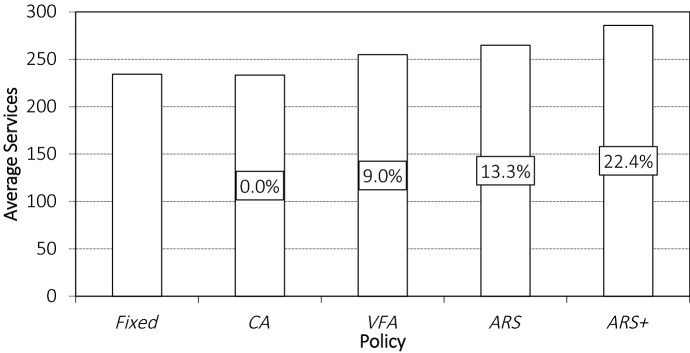
Average number of orders served and the average relative improvement over *FIXED*

To compare the policies, we calculate the (relative) improvement of a given policy over *FIXED* in terms of number of orders served, i.e., $\frac{n^{\mathbf{P }} - n^\mathbf{FIXED }}{n^\mathbf{FIXED }}$ with $n^{\mathbf{P }}$ and $n^\mathbf{FIXED }$ the number of orders served by policy $\mathbf{P }$ and *FIXED*. The number of orders served (out of an expected number of 500) and the improvement for the policies are shown in Fig. 5 (across all instance classes, i.e., across all COV values).

We observe that $$ {ARS}^+$$ provides the best solution quality with the largest number of orders serviced and the largest improvement of more than $$20\%$$. While *VFA* shows some improvement, *CA* does not. This indicates that capturing temporal dependencies and anticipating future demand are essential for good decision-making. However, initializing *VFA* with *CA* is beneficial, as *ARS* outperforms *VFA*. Finally, we see that incorporating day-of-execution corrections (when learning a policy and when executing that policy) leads to substantial performance improvement, i.e., $$ {ARS}^+$$ improves over *FIXED* by 22.4%, whereas *ARS* improves over *FIXED* by 13.3%. Thus, even a deterministic, time-dependent policy that changes the service area only a few times a day, e.g., *ARS*, already yields substantial benefits. Such policies can easily be implemented in practice as the number of service area changes is small and they occur at the same time every day.

### The value of day-of-execution corrections

In the following, we analyze the policies’ performance in more detail and focus in particular on the value of day-of-execution corrections in *ARS*$^+$.

**Fig. 6:**
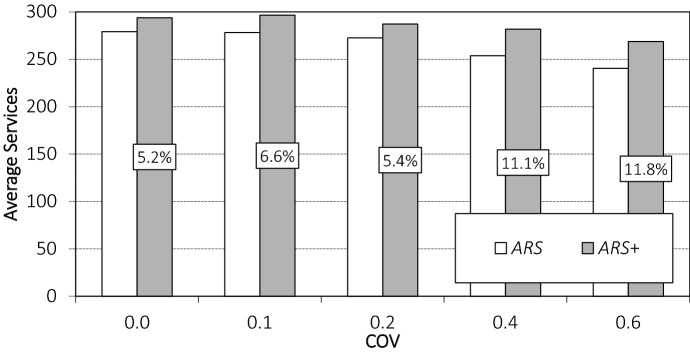
Average number of orders served for different COV values

To investigate the benefits of day-of-execution corrections, we look at the different instances classes, i.e., with different COV values, separately. Figure 6 shows the number of orders served for the different instance classes for policies *ARS* and $$ {ARS}^+$$.

As expected, the COV affects the number of orders served. For COV values greater than or equal to 0.2, when the uncertainty about the number of customer arrivals increases, the number of orders served declines. Furthermore, we see that the benefit of incorporating day-of-execution corrections increases when the uncertainty about the number of customer arrivals increases, i.e., when the day-to-day variation in number of customer arrivals is high. For COV values 0.4 and 0.6, the number of orders served when using $$ {ARS}^+$$ is more than 10% larger than when using *ARS*.

The day-of-execution mechanism is therefore particularly important when the demand volumes vary from day to day. This is also reflected in the weighting parameter of $$ {ARS}^+$$, which indicates how much emphasis is placed on the correction mechanism. For COVs of 0.0 and 0.1, the best weighting parameter is $$\alpha =0.1$$, i.e., the radii are mostly predetermined and the corrections are relatively minor. For COVs of 0.2 and 0.4, the best weighting parameter is $$\alpha =0.2$$, thus the emphasis clearly shifts toward more and more major corrections. This is even more evident for a COV of 0.6 when the best weighting parameter is $$\alpha =0.3$$.

We further analyze how the day-of-execution mechanism impacts the number of customers served and the delays. To this end, Table 2 presents average results over all COVs for the number of customers served and the average delay resulting from the five different policies. Specifically, we show the average number of customers served and its standard deviation (SD) over the 1000 days, the average delay per served customer (in minutes) and its SD over the 1000 days, and the average maximum delay per day a customer observed in minutes. It also shows the average $90\%$-percentile of the overall delay distribution in minutes, i.e., we sort the observed delays over all served customers over the 1000 simulated days in increasing order and report the delay for the customer at the $90\%$-position of all customers.

The values in the first column of the table resemble the results of Fig. 5, with *FIXED* and *CA* performing relatively poorly, *VFA* and *ARS* performing modestly, and *ARS*$^+$ clearly outperforming the other policies. As the number of customers served increases, we expect to see an increase in the standard deviation. Interestingly, the SD is smallest for *ARS*$^+$ with 43.0 even though the number of customers served is substantially more than with any other policy. The difference is largest between *ARS* and *ARS*$^+$ and the only difference between these two policies is the daily correction mechanism. The smaller SD indicates that *ARS*$^+$ better smooths the number of customers served per day even when the days differ in demand volume. A more balanced day-to-day workload has positive effects for the drivers in terms of stress and compensation. *ARS*$^+$ also has benefits for customers compared to *ARS*. Even though the average delay is slightly higher (0.93 compared to 0.92), the average SD is lower (3.07 compared to 3.41). This suggests that *ARS* has some days with several large delays and some days with hardly any delays, whereas *ARS*$^+$ adapts to the observed daily demand and smooths the delays per day. (This is also reflected in the average maximum delay.) We observe too that, every day, there is likely to be an “unlucky” customer with a delay of more than 10 minutes regardless of the policy. Similar to practice, this seems hard to avoid when serving hundreds of customers per day. However, when looking at the $90\%$-percentiles of all policies, we see that the vast majority of customers experience a delay of less than 3 minutes (if any). This shows that using a constraint in our model that limits the average delay per customer also leads to a relatively balanced distribution of delays for the customers.

**Table 2:** Policy performance

Policy	Customers	SD	Delay	Delay SD	Maximum delay	$90\%$-Percentile
*FIXED*	234.4	44.0	0.72	2.80	14.91	1.4
*CA*	233.4	44.8	0.83	3.28	18.04	1.0
*VFA*	254.9	46.7	0.89	3.19	18.42	2.2
*ARS*	264.9	49.0	0.92	3.41	20.77	2.0
*ARS*$^+$	285.3	43.0	0.93	3.07	19.78	2.8

We conclude that the newly developed day-of-execution correction mechanism does not only improve the number of customers served, but it also reduces the day-to-day variation in the number of customers served and has benefits for the experience of workforce and customers.

### Service area sizing

Next, we investigate how the different policies set the service area radius over time. To this end, we analyze the results of the instance setting with COV value 0.2 in more detail. However, the observations are similar for the other COV values.

**Fig. 7:**
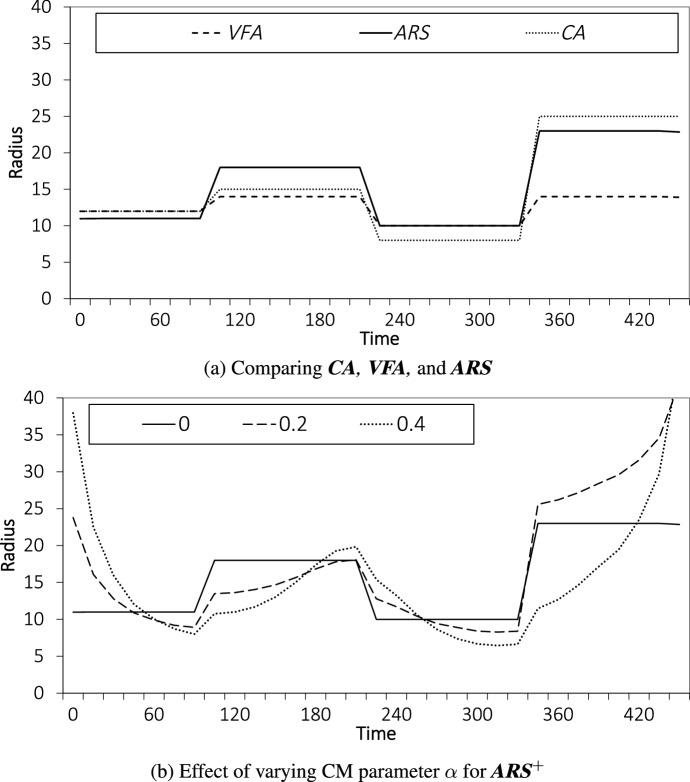
Average Radii over Time

We compare three policies: *CA*, *VFA*, and *ARS*. Figure 7a shows for each of the three policies the service area radii over the day. The service area radius changes at the start of each of the four periods (see Sect. 4). Thus, each policy adjusts the radius only three times per day.

Recalling the customer arrival distribution shown in Fig. 4, we see that the policies recognize the presence of the peaks (at 90 and at 300) and valleys by having a smaller radius in the first and third period (in anticipation of the peaks) and a larger radius in the second and fourth period of the day (in anticipation of the valleys). Furthermore, we see that initializing *VFA* with *FIXED* (giving policy *VFA*) is able to recognize the peaks, but is unable to fully capitalize on the peaks. Initializing *VFA* with *CA* (giving policy *ARS*), which already recognizes the peaks, allows *VFA* to make the necessary adjustments to better capitalize on their existence, pushing the radius out in the second and third period, but bringing the radius in in the fourth period.

It is interesting to observe that *CA* at the time of the second peak (at 300) sets a service area radius that is smaller than the other policies (even smaller than *FIXED*). The continuous approximation decides on the small radius to accommodate days with a large number of customer arrivals throughout the day to avoid the vehicles becoming too busy, which causes delivery delays. However, the results show that setting the small radius is overly conservative when customer arrivals are heterogeneous and vehicles have sufficient chance to “recover” from being busy.

Next, we analyze how incorporating day-of-execution corrections impacts the radii. We compare $$ {ARS}^+$$ for values $$\alpha = 0.0, \; 0.2$$, and 0.4 (value $$\alpha = 0.0$$ represents *ARS* (i.e., without CM)); the larger the value of $$\alpha$$, the more emphasis is placed on CM. Recall that day-of-execution corrections are considered every 15 minutes. Again, we focus on the instance setting with COV value 0.2. The results are depicted in Fig. 7b. We observe that for all values of $$\alpha$$, the radii match the customer arrival distribution with smaller radii in times of a larger number of customer arrivals. However, we also see that with CM ($$\alpha > 0$$) service area, radius changes occur more smoothly. At the start and end of the day, when the number of customers that have arrived in the last 30 minutes is small, the radii are large to ensure that vehicles are used and are not idle. It is also apparent that putting too much emphasis on corrections is undermining the value of planning. With $$\alpha = 0.4$$, we see that the policy is unable to properly anticipate. After time 300 customer arrivals start to decline and around time 360, the customer arrival peak is usually over and the radius should increase significantly. However, with $$\alpha =0.4,$$ the policy acts put too much emphasis on recent observations and keep the radius (too) small, which leads to missed opportunities and poor performance.

### Minimum service area

So far, we have assumed that the company can set the service area to any size at any time. However, in practice, there may be considerations that put limits on the service area size that can be set. For example, the company may want to offer a guaranteed service to customers living close to the facility. In the following, we analyze how enforcing a lower limit on the service area radius impacts the performance of $$ {ARS}^+$$. We denote the new policy by $$ {ARS}^+$$(*limited*) and we produce it using the exact same methodology as before.

**Fig. 8:**
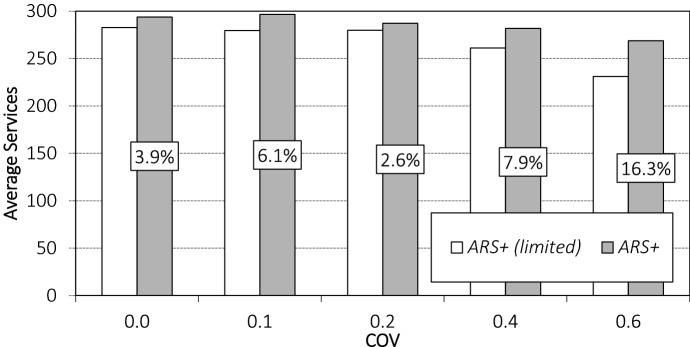
Performance of $$ {ARS}^+$$ when enforcing a minimum service area size

We compare the performance of $$ {ARS}^+$$(*limited*) to $$ {ARS}^+$$ for different COV values when we enforce a minimum service area radius equal to the service area radius of *FIXED*. The results can be found in Fig. 8.

We observe a decrease in the number of orders served when we enforce a minimum service area size. We further see that the difference between $$ {ARS}^+$$ and $$ {ARS}^+$$*(limited)* increases with increasing COV values, highlighting that guaranteeing service to a certain set of customers becomes more costly when demand is volatile.

**Fig. 9:**
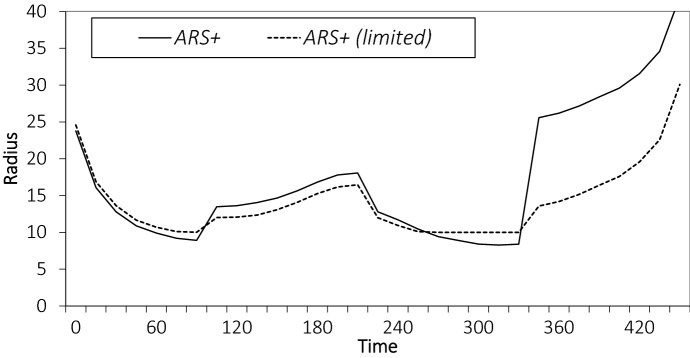
Comparison of radii over time with and without enforcing a minimum service area size

The effect of enforcing a minimum service area size can also be seen in the service area radii over time. In Fig. 9, for the instance setting with COV value 0.2, and for the variant with day-of-execution corrections, we show the radii of $$ {ARS}^+$$ and $${ARS}^+$$*(limited)*. (*FIXED* has a radius of 10 minutes for this instance setting.)

We observe that the pattern for both policies is the same. However, for $$ {ARS}^+$$*(limited)*, the radii vary less. At times with the highest number of customer arrivals (around the second peak at 300), the radii no longer drop below the minimum radius of 10. The larger radii (compared to $$ {ARS}^+$$) lead to more orders and a larger workload, thus, limiting the increase in the radius after the peak, which results in fewer orders during the period with a small number if customer arrivals.

**Fig. 10:**
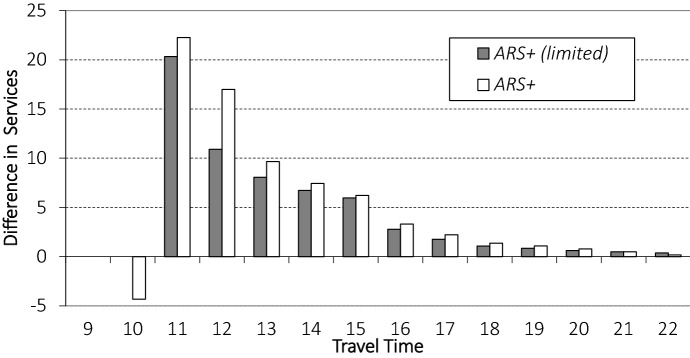
Difference in number of orders served of $$ {ARS}^+$$ and $$ {ARS}^+$$*(limited)* compared to *FIXED*

This shows that enforcing guaranteed service to a certain set of customers comes at a cost, particularly, when the number of customer arrivals is volatile. However, as we show next, even without guaranteeing service to the customer close to the facility, these customers are nearly always served. More specifically, we compare the difference in number of orders served by *FIXED*, $$ {ARS}^+$$, and $$ {ARS}^+$$*(limited)* where we partition the orders by travel time from the facility (again for the instance setting with COV value 0.2). The results are depicted in Fig. 10 where we show the relative difference to *FIXED*.

We observe that $$ {ARS}^+$$ does not serve four customers per day (on average) with a travel time to the facility of 10 (at the edge of the guaranteed service area). However, as a result, it is able to serve noticeably more orders of customers at travel times 11, 12, 13, 14, and 15 from the facility than $$ {ARS}^+$$*(limited)*. Clearly, both $$ {ARS}^+$$ and $$ {ARS}^+$$*(limited)* serve many more orders than *FIXED* (on average).

## Final remarks

We have shown how dynamically sizing the service area based on expected and observed demand can provide significant benefits for a service provider. We have also illustrated that demand volatility over time and days is an important factor when setting up and controlling the area service is offered. There are multiple avenues for future research, which we discuss briefly below.

We have focused on variation and uncertainty on the demand side, but assumed a fixed and homogeneous fleet of delivery vehicles on the supply side. Future research may focus on the supply side and investigate the benefits (and challenges) of adding drivers dynamically when demand is higher than expected. Or on supply uncertainty, which is relevant in situations where deliveries are performed by crowd-sourced drivers. Dynamically sizing the service area may be one of the mechanisms for handling supply uncertainty.

Another interesting direction, particularly in a meal delivery setting, is to investigate the interplay between different facilities (e.g., restaurants). When sharing delivery drivers across multiple facilities, sizing decisions will be interdependent. Also, customers may be served from different facilities. In such situations, sizing decisions need to be coordinated and should capture not only the expected customer arrivals but also other aspects such as fairness among the restaurants or driver familiarity with different areas of the city.

Customers expect cheap, but reliable service. Our study has shown that revenue can be increased by dynamically adjusting the service area. These benefits (or at least some) can be passed on to the customers in the form of reduced delivery prices. However, such a reduction in delivery costs would also imply a reduction in delivery reliability as service will no longer be offered at every location at any time. Dynamic pricing of service rather then dynamic availability of service may be more acceptable to customers. Therefore, a natural avenue for future research is investigating how a provider can offer service in a larger area by dynamically adjusting delivery prices.

Provide service in a larger area may also be possible by offering different service promises to different customers and, possibly, at different times. For example, customers with a travel time less than a certain threshold may be offered faster delivery than customers with a travel time that exceeds the threshold. Determining an optimal threshold in such settings is non-trivial. It should be recognized that offering customers different service promises (from different facilities) will influence customer behavior and, if not done well, may adversely affect consolidation opportunities. Because offering different service promises to different customers mainly affects the underlying routing problem, it is likely that the same techniques used to derive effective policies for service area sizing can be used to derive effective policies for service promise threshold setting.

## Footnotes

1 Such a constraint may lead to a few customers with substantial delay. However, in our computational study, the largest delay observed during a day is always less than 25 minutes and the delay for the $90\%$-quantile is between 0 and 3 minutes. Given hundreds of services per day, such values seem reasonable.

2 For the remainder of the paper, italic typesetting is used to indicate policies.
